# Clinical Evaluation of COBAS TaqMan PCR for the Detection of *Mycobacterium tuberculosis* and *M. avium* Complex

**DOI:** 10.1155/2012/170459

**Published:** 2012-09-17

**Authors:** Satoshi Ikegame, Yoritake Sakoda, Nao Fujino, Kazuhito Taguchi, Masayuki Kawasaki, Akira Kajiki

**Affiliations:** Department of Respiratory Medicine, National Hospital Organization, Omuta Hospital, 1044-1 Tachibana, Omuta-shi, Fukuoka 837-0911, Japan

## Abstract

A retrospective observational study was performed to determine the sensitivity and limitation of PCR test for the detection of *Mycobacterium tuberculosis* and *M. avium* complex. We obtained clinical specimens collected from the respiratory tract, cultured *M. tuberculosis* or *M. avium* complex, and performed PCR analysis. A total of 299 samples (*M. tuberculosis*, 177; *M. avium*, 35; *M. intracellulare*, 87) were analyzed by COBAS TaqMan PCR from April 2007 to March 2011. The PCR positivity rates were 50–55%, 70–100%, 88–98%, and 100% in smear-negative, smear 1+, 2+, and 3+ groups, respectively. The PCR positivity of tuberculosis in smear 1+ was 80.6%, which was statistically significantly (*P* < 0.001) lower than that of smear 2+ (97.3%). From January 2005 to March 2007, we collected an additional 138 samples (*M. tuberculosis*, 74; *M. avium*, 21; M. intracellulare, 43), which were analyzed by COBAS Amplicor PCR. The PCR positivity rates obtained using COBAS TaqMan PCR and COBAS Amplicor PCR were not significantly different. The sensitivity of PCR test for mycobacteria is not sufficient in case of smear 1+. Careful consideration must be given to the interpretation of negative PCR test results in smear 1+, because smear-positive tuberculosis is the criterion for isolation.

## 1. Introduction

Polymerase chain reaction (PCR), which was invented by Kary Banks Mullis [1, 2], is widely used in basic and clinical medicine. Particularly in the field of clinical medicine, PCR plays an important role in the early diagnosis of infectious diseases [3], because PCR can detect as little as one copy of DNA fragment from a pathogenic organism. The utility of PCR has also been reported in the diagnosis of mycobacteriosis [4, 5], and PCR is widely used for the detection of *Mycobacterium tuberculosis* and *M. avium* complex (MAC) in Japan. On the other hand, a specimen with a positive mycobacterial smear and a negative *M. tuberculosis* PCR test may sometimes exhibit a positive *M. tuberculosis* culture result. Such false negative results are very dangerous, because they may lead to the release of patients that pose an infection control risk. We evaluated the reliability and limitation of PCR test for the detection of *M. tuberculosis*, *M. avium*, and *M. intracellulare* in relation to the clinical situation. We found out that PCR result is sometimes not reliable in smear 1+ case.

## 2. Methods

### 2.1. Study Subjects

Clinical specimens collected from the respiratory tract (sputum or samples obtained using a bronchofiberscope (BF)) were analyzed from January 2005 to March 2011. *M. tuberculosis*, *M. avium*, or *M. intracellulare* was cultured from these specimens, and PCR of the isolated bacteria was performed at the time of sample collection.

### 2.2. Data Collection

A total of 299 samples (*M. tuberculosis*, 177; *M. avium*, 35; and *M. intracellulare*, 87) were obtained from April 2007 to March 2011, and 138 samples (*M. tuberculosis*, 74; *M. avium*, 21; and *M. intracellulare*, 43) from January 2005 to March 2007. The mycobacteria in each sample were quantified by auramine-rhodamine staining [6] and classified according to the statement of the American Thoracic Society [7].

For the assessment of false positive result, we collected 2061 samples (COBAS TaqMan PCR for *M. tuberculosis*, 1568; *M. intracellulare*, 1593; *M. avium* 1594; COBAS Amplicor PCR for *M. tuberculosis*, 505; *M. intracellulare*, 512; *M. avium* 508) in which bacterial culture showed no mycobacterial growth and PCR was checked. Samples from the patients with known mycobacterial diseases were eliminated from analysis. Briefly, false positive is defined by both negative culture result and positive PCR result in the subject without known mycobacterial diseases.

The validity and ethics of this study were approved by the Institutional Review Board (IRB) of our hospital.

### 2.3. PCR

COBAS Amplicor PCR was performed on samples collected from January 2005 to March 2007, as previously described [8, 9]. COBAS TaqMan PCR (*M. tuberculosis*, COBAS TaqMan MTB; *M. avium* and *M. intracellulare*, COBAS TaqMan MAI) was performed on samples collected from April 2007 to March 2011, as previously reported [10–12]. AMPLICOR Respiratory Specimen Preparation Kit was used for DNA extraction from clinical specimens.

### 2.4. Statistical Analysis

A chi-square test was performed to compare the positivity results between groups.

## 3. Results

### 3.1. *M. tuberculosis* PCR Positivity according to Mycobacterial Smears

Table 1 shows *M. tuberculosis* PCR positivity results from COBAS TaqMan PCR. The number of samples obtained using a BF was very small (4 samples); therefore, the total number mainly reflects sputum results. PCR positivity rates in the smear-negative, 1+, 2+, and 3+ groups were 53.1%, 80.6%, 97.3%, and 100%, respectively. PCR positivity in the smear 1+ group was statistically significantly lower than that in the smear 2+ group (*P* < 0.001, by chi-square test) and smear 3+ group (*P* = 0.006, by chi-square test). There was no statistically significant difference in the positivity between smear 1+ and tuberculosis overall (80.6% versus 86.4%, *P* = 0.36, by chi-square test).

### 3.2. *M. avium* PCR Positivity according to Mycobacterial Smears

Table 2 shows *M. avium* PCR positivity results from COBAS TaqMan PCR. The number of samples obtained using a BF was small (5 samples); therefore, the total number mainly reflects sputum results. PCR positivity rates in the smear-negative, 1+, 2+, and 3+ groups were 50.0%, 70.0%, 91.7%, and 100%, respectively. The observed trends were similar to those of *M. tuberculosis* cases.

### 3.3. *M. intracellulare* PCR Positivity according to Mycobacterial Smears

Table 3 shows *M. intracellulare *PCR positivity results from COBAS TaqMan PCR. The number of samples obtained using a BF was small (15 samples); therefore, the total number mainly reflects sputum results. PCR positivity rates in the smear-negative, 1+, 2+, and 3+ groups were 50.0%, 100%, 88.9%, and 100%, respectively. Among BF-derived samples, PCR positivity in the smear-negative group decreased to 16.7%. On the other hand, PCR positivity in the smear-positive group was 100%.

### 3.4. PCR Positivity with the COBAS Amplicor Method

Table 4 shows the results from COBAS Amplicor PCR. PCR positivity rates of *M. tuberculosis*, *M. avium*, and *M. intracellulare*, according to the quantity of mycobacteria, were almost the same as those obtained from COBAS TaqMan PCR, indicating that there were no statistically significant differences between the two PCR tests.

### 3.5. False Positive Result of the COBAS TaqMan PCR and COBAS Amplicor PCR

Table 5 shows the result of false positive result of COBAS TaqMan PCR and COBAS Amplicor PCR. False positive rate of COBAS TaqMan PCR was 6/1568 (0.38%), 3/1593 (0.19%), and 10/1594 (0.63%), in Tuberculosis, *M. avium*, and *M. intracellulare*, respectively. False positive rate of COBAS AMPLICOR PCR was 11/505 (2.18%), 2/512 (0.39%), and 1/508 (0.20%), in Tuberculosis, *M. avium*, and *M. intracellulare*, respectively.

## 4. Discussion

In this study, the sensitivity of a novel PCR method (COBAS TaqMan test) was examined by analyzing PCR positivity of clinical samples in conjunction with mycobacterial quantification by fluorescence staining. Detailed data on PCR positivity were obtained from the COBAS TaqMan test. About culture positive tuberculosis, PCR positivity in smear 1+ was 80.6%, which was statistically significantly lower than the positivity of smear 2+ (97.3%, *P* < 0.001) and smear 3+ (100%, *P* = 0.006). There was no statistically significant difference in the positivity between smear 1+ and tuberculosis overall (80.6% versus 86.4%, *P* = 0.36, by chi-square test). This indicates that we should pay careful attention to the interpretation of negative result of PCR tests in smear 1+.

PCR positivity results from COBAS TaqMan PCR are presented in Tables 1–3. The *M. tuberculosis* PCR positivity in the smear 1+ group was 80.6%, which was statistically significantly lower than that in the smear 2+ group (97.3%) (*P* < 0.001, by chi-square test). Our results indicate that the PCR result is not always accurate in smear-negative and 1+ cases. *M. avium* and *M. intracellulare* PCR positivity rates increased with the quantity of mycobacteria, as detected by fluorescent staining (Tables 2 and 3). However, PCR positivity rates of smear 1+ and smear 2+ groups were not statistically significantly different. The sample size might not be large enough to detect small differences.

There were four *M. intracellulare* PCR-negative samples in the smear 2+ group, and *M. intracellulare* PCR positivity in the smear 2+ group was 88.9%, which was slightly lower than that of *M. tuberculosis* and *M. avium*. However, the difference was not statistically significant (*P* = 0.07, by chi-square test) when compared with *M. tuberculosis* PCR positivity. Furthermore, *M. intracellulare* PCR positivity in the smear 1+ group was the same as that of *M. tuberculosis* and *M. avium*. Therefore, we concluded that the sensitivity of PCR for the detection of *M. intracellulare* was not inferior to that of *M. tuberculosis* and *M. avium*.

The number of samples obtained using a BF (4, 5, and 15 samples of *M. tuberculosis*, *M. avium*, and *M. intracellulare*, resp.) was not sufficient for analysis. The sensitivity of BF seems lower than the result of sputum overall, but PCR positivity in the smear-positive group appeared to be good.

Kim et al. [12] reported that the sensitivity of COBAS TaqMan PCR was superior to that of COBAS Amplicor PCR at the level of basic medicine. Yonemaru et al. [10] have also reported a similar superiority based on the finding that COBAS TaqMan PCR detected *M. tuberculosis* in 12 of 21 samples that were negative by COBAS Amplicor PCR. On the other hand, our results indicate that COBAS TaqMan PCR (Tables 1–3) and COBAS Amplicor PCR (Table 4) are equally sensitive. In addition, Xpert MTB/RIF [13], which detects rifampicin-resistant tuberculosis by PCR method, is reported to show higher sensitivity (smear-negative: 124/171 (72.5%), smear-positive: 551/561 (98.2%)) than the sensitivity of our study. However, a further study is required in comparing the efficacy of different PCR test, because our results are not based on the comparison of the same samples.

It may be obvious that the positivity of PCR increases along with the bacterial quantity assessed by smear. However, most previous study [10, 11, 13] did not divide smear positive cases further and analyzed overall. PCR result was thought to be very reliable in smear positive cases from these studies. In this study, we related the limitation of PCR method to the clinical situation and discovered that PCR result is sometimes not reliable in smear 1+ cases. In other words, combination of smear and PCR test would increase the sensitivity of diagnosis of tuberculosis especially in smear 1+ cases. It is very important for clinicians to determine with certainty whether mycobacteria-releasing patients have tuberculosis or nontuberculous mycobacteriosis. PCR can surely predict tuberculosis in smear 2+ or higher cases. Considerable risk exists in ruling out tuberculosis based on negative PCR results in smear 1+ cases. We must use other examinations (e.g., Quantiferon and chest CT) to make a careful decision in such cases. More sensitive methods, such as nested PCR [14], may be effective and required for smear 1+ cases.

About false positive which is defined by negative culture and positive PCR result, COBAS AMPLICOR PCR for *M. tuberculosis* showed a relatively high (2.18%) false positive rate (Table 5). COBAS TaqMan PCR and COBAS AMPLICOR PCR for *M. avium* or *M. intracellulare* showed false positive rate below 1%. Even if we excluded the case with known mycobacterial diseases from analysis, some mycobacteria (provably inactivated) might exist in the sample and make the positive PCR result. However, our retrospective study had a limitation to reveal the cause of false positive result. The cause of false positive result needs to be elucidated by the future prospective study.

The technique of PCR has progressed with time, and the speed and sensitivity have greatly improved. On the other hand, feedback from clinical medicine may not be executed in some cases. The present study is the first study to analyze the utility and limitation of COBAS TaqMan PCR for clinical use.

## 5. Conclusions

Using COBAS TaqMan PCR, we evaluated its efficacy and limitation in the context of clinical situation. The PCR positivity rate for *M. tuberculosis* in mycobacterial smear 1+ tuberculosis cases was approximately 80%. We must give careful consideration before ruling out tuberculosis based on negative PCR results, particularly when there is a small amount of bacteria in mycobacterial smears. 

## Figures and Tables

**Table 1:** *M. tuberculosis* PCR positivity rates according to fluorescent staining from Apr 2007 to Mar 2011.

Fluorescent staining	Sputum			BF			Total		
	PCR+	No	%	PCR+	No	%	PCR+	No	%
—	17	31	54.8	0	1	0	17	32	53.1
1+	29	36	80.6	0	0		29	36	80.6
2+	71	72	98.6	1	2	50.0	72	74	97.3
3+	34	34	100	1	1		35	35	100

Total	151	173	87.3	2	4	50.0	153	177	86.4

G: Gaffky scale.

BF: bronchofiberscopy.

**Table 2:** *M. avium* PCR positivity rates according to fluorescent staining from Apr 2007 to Mar 2011.

Fluorescent staining	Sputum			BF			Total		
	PCR+	No	%	PCR+	No	%	PCR+	No	%
—	4	7	57.1	1	3	33.3	5	10	50.0
1+	5	8	62.5	2	2	100	7	10	70.0
2+	11	12	91.7	0	0		11	12	91.7
3+	3	3	100	0	0		3	3	100

Total	23	30	76.7	3	5	60.0	26	35	74.3

G: gaffky scale.

BF: bronchofiberscopy.

**Table 3:** *M. intracellulare* PCR positivity rates according to fluorescent staining from Apr 2007 to Mar 2011.

Fluorescent staining	Sputum			BF			Total		
	PCR+	No	%	PCR+	No	%	PCR+	No	%
—	10	16	62.5	1	6	16.7	11	22	50.0
1+	14	14	100	3	3	100	17	11	100
2+	26	30	86.7	6	6	100	32	36	88.9
3+	12	12	100	0	0		12	12	100

Total	62	72	86.1	10	15	66.7	72	87	82.8

G: gaffky scale.

BF: bronchofiberscopy.

**Table 4:** *M. tuberculosis*, *M. avium*, and *M. intracellulare* PCR positivity rates according to fluorescent staining from Jan 2005 to Mar 2007.

Fluorescent staining	*M. tuberculosis*				*M. avium*				*M. intracellulare*			
	PCR+	No	%	P*	PCR+	No	%	P*	PCR+	No	%	P*
—	12	18	66.7	0.61	4	7	57.1	0.84	10	12	83.3	0.12
1+	10	12	83.3	0.83	4	4	100	1.00	7	9	77.8	0.21
2+	22	23	95.7	0.36	7	7	100	0.78	14	15	93.3	0.98
3+	21	21	100	1.00	3	3	100	1.00	7	7	100	1.00

Total	65	74	87.8		18	21	85.7		38	43	88.4	

G: gaffky scale.

*PCR positivity was compared with the corresponding columns in Tables 1–3.

**Table 5:** False positive result of COBAS TaqMan PCR and COBAS AMPLICOR PCR.

	Tuberculosis	*M. avium*	*M. intracellulare*
COBAS TaqMan PCR	6/1568	3/1593	10/1594
FP/total number	(0.38%)	(0.19%)	(0.63%)
COBAS AMPLICOR PCR	11/505	2/512	1/508
FP/total number	(2.18%)	(0.39%)	(0.20%)

FP: false positive.
